# Tip of the Iceberg? Country- and Company-Level Analysis of Drug Company Payments for Research and Development in Europe

**DOI:** 10.34172/ijhpm.2022.6575

**Published:** 2022-03-15

**Authors:** Piotr Ozieranski, Luc Martinon, Pierre-Alain Jachiet, Shai Mulinari

**Affiliations:** ^1^Department of Social and Policy Sciences, University of Bath, Bath, UK.; ^2^Euros for Docs, Paris, France.; ^3^Department of Sociology, Lund University, Lund, Sweden.

**Keywords:** Pharmaceutical Industry, Payments, Research and Development, Transparency, Conflict of Interest, Financialization

## Abstract

**Background:** Creating new therapies often involves drug companies paying healthcare professionals and institutions for research and development (R&D) activities, including clinical trials. However, industry sponsorship can create conflicts of interest (COIs). We analysed approaches to drug company R&D payment disclosure in European countries and the distribution of R&D payments at the country and company level.

**Methods:** Using documentary sources and a stakeholder survey we identified country- regulatory approaches to R&D payment disclosure. We reviewed company-level descriptions of disclosure practices in the United Kingdom, a country with a major role in Europe’s R&D. We obtained country-level R&D payment data from industry trade groups and public authorities and company-level data from eurosfordocs.eu, a publicly available payments database. We conducted content analysis and descriptive statistical analysis.

**Results:** In 32 of 37 studied countries, all R&D payments were reported without named recipients, following a self-regulatory approach developed by the industry. The methodological descriptions from 125 companies operating in the United Kingdom suggest that within the self-regulatory approach companies had much leeway in deciding what activities and payments were considered as R&D. In five countries, legislation mandated the disclosure of R&D payment recipients, but only in two were payments practically identifiable and analysable. In 17 countries with available data, R&D constituted 19%-82% of all payments reported, with self-regulation associated with higher shares. Available company-level data from three countries with self-regulation suggests that R&D payments were concentrated by big funders, and some companies reported all, or nearly all, payments as R&D.

**Conclusion:** The lack of full disclosure of R&D payments in countries with industry self-regulation leaves considerable sums of money unaccounted for and potentially many COIs undetected. Disclosure mandated by legislation exists in few countries and rarely enhances transparency practically. We recommend a unified European approach to R&D payment disclosure, including clear definitions and a centralised database.

## Background

**Key Messages**

** Implications for policy makers**

- In European countries, drug company payments to healthcare professionals and institutions for research and development (R&D), including clinical trials, involve vast amounts of money.
- However, self-regulation of disclosure of R&D payments by drug companies leaves recipients of these crucial payments unknown.
- In interpreting self-regulatory rules, industry trade groups and companies have much leeway in deciding what counts as R&D and therefore may potentially inflate the value of payments without disclosing their recipients.
- A few countries introduced legislation mandating the disclosure of R&D payment recipients, but payment data was typically presented in ways that make it difficult for the public and researchers to analyse it.
- All drug company R&D payments must be clearly defined and disclosed on a name basis in easily accessible, searchable, and analysable databases to ensure transparency of R&D activities and protect public health from any bias that could result from the undisclosed receipt of such payments.

** Implications for the public**

Creating new therapies often involves drug companies paying healthcare professionals and institutions for research and development (R&D) activities, including clinical trials. Although R&D payments are worth billions of euros, it remains unknown who receives them in most European countries. Consequently, we cannot tell whether such payments create conflicts of interest (COI) that may affect the actions of those who receive them in ways potentially compromising good scientific practice or patient care. We explain how payments for R&D activities could be made more transparent by creating an easily accessible, searchable online database that anyone could use to check the details of companies making R&D payments, their recipients, payment value and purpose. On its own, increased transparency of R&D payments is unlikely to prevent bias and unethical behaviour, where such exist. Nevertheless, transparency is vital for understanding financial relationships between drug companies, researchers and research institutions and developing complementary policies.

Analyses of the structure of the modern-day pharmaceutical industry traditionally distinguish a consistent core of large-scale multinational corporations (“big pharma”) with diverse product portfolios and an increasing number of smaller biotechnology companies creating drugs from living cells (“biologics”).^1-4^ While the sector’s recent evolution has blurred some of the differences between the two segments,^3,4^ their key distinguishing feature is the focus on research and development (R&D), the process of discovering, developing, and testing new drugs with a view to obtaining regulatory approval and market access.^1,2,5^ This process starts with basic research, drug discovery, pre-clinical testing, through to phase I-III clinical trials,^2,6^ but some also see the “pre-approval R&D” as complemented by “post-approval” R&D work on new indications or phase IV trials.^7^ The R&D process involves extensive collaboration between the industry academia, government and regulatory bodies, with key sources of investment including the industry, philanthropic organisations, and government, the latter particularly in relation to basic research.^2,8,9^

 What demonstrates pharmaceutical companies’ key role in bringing drugs to the market^10,11^ are rapidly escalating R&D costs,^12^ reaching, according to an industry-supported research centre, $2.6bn per drug.^7^ These cost calculations help the industry justify the need for longer patent and market protection for drugs^13,14^ or their high prices, especially in the US.^15-18^

 Nevertheless, the estimates of the high cost of industry R&D have been challenged,^19,20^ including a recent alternative of $0.6bn per drug.^21^ In fact, R&D investment is reduced following the increasing “financialization” of big pharma.^17,22,23^ For example, a US Congress investigation established that “From 2016 to 2020, the 14 leading drug companies spent $577 billion on stock buybacks and dividends—$56 billion more than they spent on R&D over the same period.”^24^ Further, illustrative data from the US suggests that the drive towards revenue maximisation is associated with spending on medical marketing increasing from $17.7 to $29.9 billion from 1997 to 2016.^25^ Similarly, the pharmaceutical and health product industry seek to create a policy environment conducive to protecting their revenues by spending on lobbying and political campaigns, totalling nearly $6 billion between 1999 and 2018.^26^ In the context of drug development, securing revenue streams involves companies prioritising slight modifications of existing drugs, often offering minor therapeutic advances, over risky investments into new innovative drugs.^13,3,27,28^ Given the lower costs and investment risks, promising compounds are often obtained through mergers and acquisitions, especially of biotech companies.^17,22,23,29^ For similar reasons, financialization has increased the outsourcing of drug companies’ in-house R&D, sometimes all its phases.^12,23^ The outsourcing typically entails working with contract research organisations (CROs) which manage links with research partners.^12,23,28^ Companies also establish partnerships with and commercialise discoveries made at publicly funded academic research centres, which is where most of the innovative molecules originate.^22,23,29-31^

 The trends towards maximising shareholder value and minimising investment risks brought in by financialization exacerbate the well-documented tension between commercial profitability and public health objectives in the provision of safe, effective, and affordable medicines.^32-35^ One key area of concern is conflicts of interest (COIs) that may arise in the industry’s research collaborations with hospitals, universities, as well as individual scientists and clinicians. Although these collaborations have brought in many breakthrough therapies, COIs associated with industry sponsorship of R&D may bias study design, conduct, outcomes, and dissemination.^36-40^ For example, financialization reinforces concerns about COIs caused by the blurring of divisions between academia and corporations.^41^ Similar criticisms have been made regarding COIs associated with collaborations managed by some CROs.^28,42^

 Over the last decade a major global trend in addressing these concerns has involved disclosure of industry payments to healthcare professionals and organisations, including those involved in research activities.^43-47^ For example, the US Sunshine Act currently mandates pharmaceutical and medical device companies to publish, in the Open Payments database, payments exceeding $10 made to physicians and teaching hospitals.^44,48^ There has been increasing scrutiny of so-called general payments, such as “honoraria, gifts, meals, consulting fees, and travel compensation,”^49^ including those made in relation to research. For instance, receiving general payments by authors was associated with positions favourable to the funding companies expressed in editorials, guidelines and review articles.^50^ While some studies have documented an association between general payments,^51^ including consultancies,^52^ and positive clinical trial outcomes, others have established no such relationship.^53^ Further, undisclosed general payments, detected by comparing Open Payments with researchers’ self-reporting, are widespread in articles presenting findings from research studies,^54-56^ including reports of clinical trials.^57^

 Less attention has been given to actual “research payments” – “direct compensation to physicians, funding for research study coordination and implementation” reported in Open Payments.^58^ This is consistent with their lower prevalence than general payments among payments to physicians,^57,59,60^ who have been the focus of Open Payments scholarship. Only few specialties, including ophthalmology^61^ as well as haematology and oncology,^62^ have recorded more prevalent research payments. However, with some exceptions,^57^ in most specialties and professional roles, research payments have higher values than general payments.^59,60,63,64^ Research payments are typically concentrated in a few top funders^59,65^ and recipients.^65-68^ Indications exist that they are often made for clinical trials,^65^ especially those involving the funder’s drug.^69^ Research payments are associated with higher professional experience and academic leadership^65^ as well as academic influence.^70,71^ However, their impact on research results is unclear, with one study finding no significant relationship between company-funded grants and clinical trial outcomes.^52^ Further, sometimes undisclosed research payments are more common than undisclosed general payments,^72^ but the opposite has also been observed.^55,57,73^

 In addition to research payments made to individual physicians, Open Payments covers payments for “associated research,” including grants,^57^ made to “a research institution or entity where a physician is named as a principal investigator on the research project.”^74^ Payments for associated research have attracted even less scrutiny than research payments, consistent with the exclusion of research institutions, such as universities, from the US Sunshine Act disclosure requirements.^48^ Nevertheless, in some specialties, such as neurology^54^ or oncology,^57^ the value of organisational-level payments for associated research has exceeded individual-level research payments. In these specialties, undisclosed payments for associated research were also worth more than undisclosed research payments.^54,57^

 Unlike the United States, most European countries follow a self-regulatory approach to payment disclosure, with minimum standards included in the Code of Practice of the European Federation of Pharmaceutical Industries and Associations (hereafter “EFPIA Code”) and adopted in the codes of practice of EFPIA’s national member associations.^75,76^ These provisions are binding for their company members and non-member associations and companies following them voluntarily.^75^ Only countries which EFPIA recognises as having legal or other self-regulatory requirements equivalent to the EFPIA Code are exempted from its application.^76,77^

 The EFPIA Code stipulates that research payments, termed “R&D payments,” made to individuals or organisations be disclosed in relation to clinical and non-clinical studies and prospective non-interventional studies.^75^ However, companies report them as lump sums, without named recipients.^75,78^ This uninformative form of disclosure is compounded by the low accessibility of payment data in many countries, with disclosures published on individual company websites in the portable document format, severely undermining possibilities for analysis.^43,76,79,80^ These challenges in data presentation may explain why – except for one study of Ireland – R&D payments have escaped research scrutiny.^78^

 As self-regulation is implemented without legal sanctions, it remains unknown how faithfully the provisions regarding the reporting of R&D payments are interpreted by trade groups and companies. EFPIA expects its member associations to establish complaints procedures and sanctions for breaching industry codes and publishes annual reports summarising implementation activities undertaken by member associations.^77,81^ However, studies of the self-regulation of drug promotion have documented significant discrepancy between industry codes and the actual conduct of the industry or specific companies.^79,82-84^

 It is unclear to what extent the limited disclosure of R&D payments within self-regulation has been addressed by the “Sunshine” legislation or other regulatory interventions introduced in several European countries.^43-45^ Nevertheless, the examples of Spain and the Netherlands, where R&D payments have been exempted from mandatory disclosure on a name basis,^43^ suggest limited transparency gains compared to self-regulation.

 However, shedding more light onto R&D payments has recently been enabled by country-level annual payment summaries published as part of EFPIA’s compliance monitoring.^85^ Further, eurosfordocs.eu,^86^ a new database integrating payments from eleven countries, has enabled comparative company-level analysis.^76,79^ Finally, “methodological notes” accompanying company disclosures in countries with self-regulation^78,87^ may illuminate how the industry interprets code provisions regarding, for example, the demarcation of R&D from non-R&D payments, which should be reported on a name-basis (subject to recipient consent in most countries).^43^

 We aim to, first, map regulatory approaches to R&D payment disclosure in Europe. Second, explore, using a country case study, how the scope of R&D payment disclosure is shaped by the practices of trade groups and companies which implement EFPIA’s self-regulatory rules. Third, compare the scope of disclosure of R&D payments in countries with disclosure mandated by public regulation. Fourth, examine the country- and company-level distribution of R&D payments in European countries with available data.

## Methods

###  Data Collection 

 We mapped approaches to payment disclosure in European countries by surveying available academic literature in Scopus (search terms: “Sunshine Act,” “Open Payments,” “European Federation of Pharmaceutical Industries and Associations” or “EFPIA” and “disclosure”). The same strategy involved using google searches to find grey literature. Additionally, searches on EFPIA’s website and the websites of its trade group members identified their codes of practice, reports, and press releases relating to payment disclosure. We triangulated these sources using country profiles compiled by MediSpend^88^ and company disclosure reports and methodologies from the websites of four major companies present in most European countries (Amgen, GSK, Merck Serono, Bayer). In countries with public regulation, we examined legislation, decisions by data protection agencies, legal commentary, websites of regulatory bodies involved in payment disclosure, and disclosure databases. Finally, between mid-November 2020 and January 2021, we conducted an email survey (see Supplementary file 1). Of 34 approached pharmaceutical industry trade groups, 17 responded, including 14 which answered at least some of the questions. Of 13 approached public or self-regulatory/public bodies, ten replied. Of those, six answered at least some of the questions, three sent holding messages, and one redirected us to another institution. Exceptionally, where data was not available in English we translated it with Google Translate and Deepl.com, and, if necessary, sought clarification from the survey respondents and colleagues with the knowledge of local languages.

 To identify provisions pertaining directly to R&D payment disclosure we examined the EFPIA Code (countries with self-regulation) as well as legislation, regulatory decisions by data protection agencies, regulatory bodies’ websites, and disclosure databases (countries with public regulation or combining public and self-regulation). We conducted a case study of the United Kingdom to explore how EFPIA member trade groups and drug companies interpret the EFPIA Code. Using the terms “research and development” and “R&D” we extracted information from the Code of Practice of the Association of the British Pharmaceutical Industry^89^ (hereafter: “ABPI Code”) and methodological notes published in 2019 by 125 companies participating in Disclosure UK, a self-regulatory disclosure platform for ABPI members and other companies following the ABPI Code voluntarily.^90^ Our choice of the United Kingdom recognised its key position in pharmaceutical industry R&D in Europe^91^ and comparatively high R&D payment shares,^81^ which should allow for capturing varying company disclosure practices. We also investigated whether evidence existed of monitoring of compliance with the ABPI Code’s disclosure requirements. In 1993, the ABPI established the Prescription Medicines Code of Practice Authority (PMCPA) as the quasi-autonomous body responsible for administering its Code.^84^ The PMCPA publishes reports of all cases it considers on its website irrespective of the verdict.^92^ The case reports can be searched using the specific clauses of the ABPI Code that are cited. Following an earlier analysis of PMCPA investigations,^82^ we collected all case reports that involved alleged or confirmed breaches of disclosure requirements, ie, clauses 21 and 24, and checked whether any pertained to R&D payment disclosure.

 We extracted country-level R&D payment data separately in countries following the EFPIA-recommended “disclosure template”^75^ and in countries with unique payment categories introduced by legislation. Within the former group of countries, we considered a 2019 Europe-wide report collating data from EFPIA trade group members.^81^ We sought for any missing information in trade group annual reports and press releases. We also extracted information from databases run by the trade groups in Romania (R&D payments)^93^ and the United Kingdom (all payments).^94^ Finally, using eurosfordocs.eu we generated summaries of R&D and non-R&D payments reported in Belgium, Ireland, and non-R&D payments in Romania.^95^ Finally, we extracted payment data from the responses to the stakeholder survey.

 We were able to collect country-level R&D data for two countries with unique payment categories introduced by legislation – Slovakia and France. The French data was extracted from eurosfordocs.fr, a database run by a non-governmental organisation enhancing the accessibility of payments reported in a government-run payments database, Transparence Santé. The Slovak data was extracted from a database published by Národné Centrum Zdravotníckych Informácií.

 Finally, we used eurosfordocs.eu to extract company-level data from three countries, Belgium, Ireland, and the United Kingdom, disclosing R&D payments following EFPIA’s disclosure template and having complete data for all companies.^79^ To account for year-to year payment variability and multi-year payments, especially relevant for clinical trials, we considered the entire 2017-2019 data series covered by eurosfordocs.eu. We contextualised company payment data using global company sales values available from the corresponding yearly rankings of the 50 world’s largest companies.^96-98^ We also reviewed the drug and clinical trial portfolios reported on the websites of the top three companies with the highest R&D payment shares in each country.

###  Analysis

 We categorised and compared regulatory approaches to R&D and non-R&D payment disclosure as self-regulation or public regulation. Our coding drew on earlier research,^43,76^ while replacing the “government regulation” category with “public regulation,” comprising legislation (eg, “Sunshine Acts”) and regulatory interventions by data protection agencies clarifying the interpretation of existing data privacy laws regarding payments drug company payments.

 Building on European research on disclosure data,^78,87^ we coded the methodological notes of Disclosure UK participants to establish whether these documents mentioned R&D or provided its definition. A separate inductive categorisation captured specific forms of R&D payments and study types mentioned by companies in addition to, or instead of, the EFPIA or ABPI R&D definition. These codes focused on capturing disclosure practices shared by the greatest possible number of companies. Regarding countries with public regulation, inductive codes captured how the scope of R&D payment disclosure was shaped directly and indirectly by legal provisions. All content analysis was conducted by one researcher and validated by discussions within the research team. Any differences were resolved by agreement.

 One researcher analysed country-level payment data descriptively in MS Excel by calculating the shares of R&D payments within all payments and by dividing R&D payments by each country’s population size. The largest funders for the 2017-2019 period were identified using the interquartile range and the Gini index, a relative measure of payment concentration, with values ranging from 0 (all companies make equal payments) to 1 (one company makes all payments).^87^

## Results

###  The Make-up of European Regulatory Approaches to R&D Payment Disclosure 

 R&D payments are disclosed following the EFPIA Code^75,99^ in 33 of the 37 studied European countries (Table 1).

**Table 1:** Regulatory Approaches to Disclosure of R&D Payments in Europe

**Country**^a^	**Self-regulation**^b^	**Public Regulation**^c^
Austria	✓	
Bosnia & Herzegovina	✓	
Belgium	✓^d,e^	
Bulgaria	✓	
Croatia	✓	
Cyprus	✓	
Czech Republic	✓	
Denmark		✓^f^
Estonia	✓^d^	
Finland	✓^d^	
France		✓
Germany	✓	
Greece	✓^d^	
Hungary	✓^d^	
Iceland	✓	
Ireland	✓	
Italy	✓	
Latvia	✓^d^	
Lithuania	✓^d^	
Luxembourg	✓	
North Macedonia	✓	
Malta	✓	
The Netherlands	✓^d,e^	
Norway	✓	
Poland	✓	
Portugal		✓
Romania	✓^d^	
Russia	✓	
Serbia	✓	
Slovakia		✓^g^
Slovenia	✓	
Spain	✓^d,e^	
Sweden	✓	
Switzerland	✓	
Turkey		✓
UK	✓	
Ukraine	✓	
n = 37	n = 32	n = 5

Abbreviation: R&D, research and development. ^a^ Countries excluded from analysis are Albania, Andorra, Belarus, Lichtenstein, Monaco, Montenegro, San Marino, and Vatican City. ^b^ Self-regulation of R&D payment disclosure in all studied countries is based on the EFPIA Code. In the column listing countries with self-regulation, it applies to the disclosure of all R&D payments. Therefore, this column excludes Slovakia, where only some R&D payments are disclosed under self-regulation. ^c^ Public regulation of payment disclosure in all studied countries is based on legislation. ^d^ Countries with self-regulation of R&D payment disclosure and public regulation of non-R&D payments. In all these countries, the public regulation of non-R&D payments is based on legislation. In addition, Greece also has public regulation in the form of a clarification decision issued by the data protection agency. ^e^ In addition to public regulation, the regulation of non-R&D payments in Belgium, Spain and the Netherlands is combined with elements of self-regulation. In Belgium and the Netherlands, the online disclosure platforms are managed by multi-stakeholder bodies, including the pharmaceutical industry. In Spain, payment disclosure is managed by individual drug companies, and overseen by the pharmaceutical industry trade group, without involvement from public authorities. ^f^ In Denmark, R&D payments are part of individual-level payments called “professional affiliations,” which are disclosed, together with individual-level payments for conference participation abroad, based on public regulation taking the form of legislation. Separately, the Danish pharmaceutical industry trade group discloses non-R&D organisational-level grants to hospitals using its own self-regulatory code which is separate from the provisions of the EFPIA Code. ^g^ In Slovakia, R&D and non-R&D payments made to individual-level “healthcare workers” and organisational-level healthcare providers are disclosed based on public regulation. However, R&D and non-R&D payments to organisational-level non-healthcare providers fall under self-regulation.

 In 22 of the 32 countries, self-regulation is the sole approach to both R&D and non-R&D payment disclosure. It is managed by EFPIA member trade groups and, in Luxembourg, a non-EFPIA member association voluntarily committing to observe the EFPIA Code.^100^

 In ten further countries, self-regulation applies to the disclosure of all R&D payments, while public regulation covers at least some non-R&D payments. In Belgium,^101^ Greece^102-104^ and Romania,^105,106^ legislation states that R&D payments be disclosed under the EFPIA Code. Similarly, in Spain^107^ and the Netherlands,^108,109^ data protection agencies have clarified that all non-R&D payments can be lawfully disclosed, including named recipients, without introducing new legislation. However, the Estonian,^110,111^ Hungarian,^112,113^ and Latvian^114,115,116^ disclosure legislation focuses on payments for event participation, and, in Lithuania, also on fees for service.^81,116-118^ In these countries, therefore, the disclosure of R&D payments is implicitly left to the EFPIA Code. Likewise, in Finland, legislation has introduced mandatory payment disclosures only for healthcare organisations, yet without referring to R&D payments.^119^

 Only five countries have public regulation mandating disclosure of at least some R&D payments. In France, Portugal, and Turkey legislation is the sole approach applicable to all R&D and non-R&D payments. In Denmark, the disclosure of all R&D payments falls under public regulation. In addition, non-R&D grants to hospitals are disclosed via a self-regulatory code,^120^ but all other individual-level payments (called “professional affiliations” and “payments for conference abroad”) are disclosed via public regulation.^121^ Finally, in Slovakia, public regulation covers certain R&D and non-R&D payments made to individual-level “healthcare workers” and organisational-level healthcare providers.^77,122^ Payments from outside of this list and those made to organisational-level recipients which are not healthcare providers, but otherwise meet EFPIA’s definition of the “healthcare organisation,” are disclosed under the EFPIA Code.^103,105^

###  Scope of Disclosure – Industry Self-regulation

 In countries disclosing R&D payments following the EFPIA Code, they are presented as the country total per company of all payments to individuals and organisations (Section 23.05). The definition of studies in relation to which R&D payments are made (Annex B in the EFPIA Code) reflects the Organisation for Economic Co-operation and Development Principles on Good Laboratory and Practice and the European Union (EU) Directive 2001/20/EC on Clinical Trials (superseded by EU Regulation N°536/2014). This definition involves the “planning” and “conduct” of non-clinical (eg, laboratory) studies, clinical trials (phase I-IV), and *prospective* non-intervention studies (studies involving medicines being “prescribed in the usual manner in accordance with the terms of the marketing authorisation”).^75^ However, payments associated with *retrospective* non-intervention studies (eg, reviews of records or databases^75^) should be recorded within two other, non-R&D, payment categories published on a name basis subject to recipient consent – contributions to costs of events or fees for service or consultancy.^75^ The EFPIA Code stipulates that non-intervention studies (*prospective* or *retrospective*) “that are not conducted to maintain a marketing authorisation” (in application and following definitions of the “Clinical Trials” Regulation 536/2014), will be disclosed under “consultancy/fee-for-services.”^75^

 The EFPIA Code does not specify *activities* associated with the three study types defined as R&D. Instead, it allows companies to consider “costs related to events that are *clearly related to*” R&D activities (emphasis added) as R&D payments,^75^ rather than reporting them as non-R&D “contributions to costs of events.” Additional explanatory notes state that R&D payments may include “agreement for delivering clinical trials”^123^ without specifying which consultancies can be disclosed as R&D payments. Therefore, the exact scope of R&D payment reporting is determined by EFPIA’s member trade groups and their company members, which “are encouraged to include a comment in the Methodological Note, where appropriate”^75^ to explain how they interpret and operationalise the EFPIA Code.

 We illustrate this process using the UK as an example. The ABPI Code restates EFPIA’s R&D definition (Clause 23.2). The ABPI Code stipulates that R&D may involve payments to consultants, including for clinical trials (Clause 23.2). However, consultancy payments for market research (defined as “collection and analysis of information” regarding drugs – Clause 12.2) are reported either as non-R&D fees for service and consultancy (when the company knows the identities of research participants) or not disclosed at all (when participant identities are unknown) (Clause 23.3). Nevertheless, costs *related to* R&D activities seem broadened compared to the EFPIA Code, as they *can* include costs “subsidiary to these activities” (Clause 23.2).

 The ABPI Code is interpreted by each Disclosure UK participant, including both ABPI members and many non-members following it voluntarily. Of 125 companies submitting their methodological notes alongside payment disclosures in 2019, 83 (66%) mentioned “research and development” or “R&D.” Of those, 54 (65%) repeated the EFPIA/ABPI definition of R&D or stated relevant provisions of the EFPIA/ABPI Codes. Of the remaining 29 companies which referred to R&D but not the EFPIA/ABPI definition, 8 (28%) noted only one or two of the three study types covered by these definitions, primarily clinical studies. However, in all but one cases it was unclear whether no payments relating to the omitted study types had been made or whether these payments had been made but not reported.

 Of the 83 companies mentioning R&D, 66 (80%) offered additional information instead of, or in addition to, the EFPIA/ABPI definition in relation to the *forms of payments* and *study types* associated with R&D.

 The *forms of payments* mentioned most frequently (upper half of Table 2) were fees for service and consultancy (35% – hereafter the denominator is the 66 companies providing additional information on R&D payments). Some companies defined them narrowly, for example, as investigator fees. Others, however, also mentioned fees for various meetings considered “relevant” for conducting the studies covered by EFPIA’s R&D definition. Some companies went further by including speaker and meetings fees, which, arguably, might have been reported as non-R&D fees for service and consultancy. Some differences existed in the scope of R&D payments linked to consultancy. For example, although Otsuka reported “support (…) to medical publication in connection to R&D activities” under R&D, Baxter stated that “Medical writing (unless the medical writing forms an integral part of an Investigator Initiated trial)” would be disclosed under non-R&D fees for service and consultancy.

**Table 2:** Disclosure Practices Relating to R&D Payments by Companies Following the ABPI Code (2019)

**Forms of Disclosed R&D Payments**		
**Fees for Service and Consultancy**	**Meetings and Events**	**Grants**
“Institution and Investigator Agreements” for clinical trials (Mitsubishi Tanabe) “[I]nvestigator fees” (Grünethal, Daiichi Sankyo) “Advisory Boards and consultancy services” (Chugai, Immedica, Sobi) “Ethics committee fees” (Ipsen) “Consultancy activities related to…steering committee… activities” (Mitsubishi Tanabe) “Data Monitoring Committees” (Pfizer) “Speaker agreements” (Mitsubishi Tanabe) or “programs” (Novartis, Sandoz) “Consultancy activities related to…scientific meetings” (Novartis, Sandoz)	“Costs related to events that are considered essential to effective study conduct eg, Investigator Meetings, Steering Committee Meetings, Data Monitoring Committees” (Amgen) “For authors/presenters of abstract/poster connected to a Trial/Study/Project ID, the registration fee is disclosed under R&D” (Novo Nordisk) “[T]ravelling expenses for HCPs involved in clinical trials to travel to meetings” (Santhera) “[I]nvestigator meetings … incl. infrastructure, travel, logistic and with exclusion of meals whenever possible” (Novartis, Sandoz)	“Grants – where unrestricted funds are given to a HCP or HCO explicitly for the purposes of R&D work – are also included in the aggregate” (Clinuvel) “Independent Medical Grants” (Pfizer) “Grants” (Pharmamar)
**Study Types Mentioned in Company Methodological Notes but not in EFPIA/ABPI Definition of R&D**		
**“Research,” Including “Basic Research” and “Research Collaborations”**	**Investigator Sponsored Studies or Investigator Initiated Studies (Part of Prospective Non-interventional Studies or Clinical Research)**	**Real World Data Studies and Health Outcomes Research**
“We publish the total value of ToV for basic research under the category ‘R&D’” (Grünethal) “As basic research is usually targeted at either developing new products or relates to a specific product and is intended to extend its scope of use, we will publish the total value of ToV under the category ‘R&D’” (Bayer) “[E]arly-stage research” (Novartis, Sandoz) “Clinical & Research Collaboration” (Pfizer, Daichii Sankyo) “Further research into the fields relevant to CLINUVEL’s development of its products” (Clinuvel)	“Investigator initiated and sponsored Studies” (Shire) “[R]esearch may be undertaken by individual HCPs and/or HCOs where they would like to investigate a particular aspect of a Gilead/Santhera medicine. This type of research, where supported by paying a Grant to the relevant HCO, is reported as a ToV under the appropriate heading” (Gilead, Santhera) “Sobi will include ToVs related to Sobi-sponsored studies as well as non-Sobi-sponsored studies in the R&D category” (Sobi) “Pre-clinical research and clinical research (includes ISR)” (Chugai) “[A]ny ToV relating to prospective non interventional studies sponsored by investigator (Eg, ISS), as they are prospective in nature” (Otsuka)	“Real world data studies and Health Outcomes research” (Chugai, Immedica) “ToVs related to licensing fees paid for the use of Clinical/Health Economics and Outcomes Research questionnaires and tools, if the questionnaires and tools are intended for use with a Research and Development project/study are reported in aggregate form under the ‘Research and Development’ category” (Novartis, Sandoz)

Abbreviations: ABPI, Association of the British Pharmaceutical Industry; R&D, research and development; ISR, Investigator Sponsored Researchl; ISS, Investigator Sponsored Studies; EFPIA, European Federation of Pharmaceutical Industries and Associations; HCPs, healthcare professionals; HCO, healthcare organisation; ToV, transfer of value (ie, payment). *Notes:*The company methodological notes corresponding with the quotes in the table can be accessed via the Disclosure UK website.^124^ The table shows typical examples of payment and study types where agreement existed between companies. Some of the companies with reported quotes (eg, Novartis) are parents and others (eg, Sandoz) are subsidiaries publishing the same methodological notes.

 The second most frequently mentioned payment form (30%) were costs of event participation. As with consultancy payments, some companies mentioned meetings deemed “essential” for R&D activities (eg, Amgen), while others referred to them as “associated with” (ApoPharma) R&D activities. Further, while some companies listed meetings relevant for the study “conduct,” others also mentioned those involved in dissemination of findings, which ordinarily would be disclosed as non-R&D “contributions to costs of events.” However, for Bristol Myers Squibb, supporting attendance at “scientific congresses” was “not *generally* considered R&D expenditure” (emphasis added), and Novo Nordisk similarly restricted sponsorship for “passive” delegates.

 The third most frequently mentioned payment form (9%) was grants. While some companies (eg, Clinuvel) stressed that grants had to be “explicitly” linked to R&D, others left unexplained the rationale behind not reporting them as non-R&D “grants and donations.” Some (eg, Santhera) stated, consistent with the ABPI Code (clause 24.2), that grants were addressed *only* to organisations. However, others did not clarify grant recipients (eg, Pfizer) or mentioned both organisations and individuals (eg, Clinuvel).

 The additional information provided in some methodological notes also described *study types* not listed explicitly in EFPIA’s R&D definition (bottom half of Table 2). Most companies (33%) referred to Investigator Sponsored Studies or Investigator Initiated Studies. These studies were often placed under prospective non-interventional studies, covered by EFPIA’s R&D definition (eg, Otsuka), mentioned as pre-clinical or clinical research (eg, Immedica), or a stand-alone study type (eg, CSL Behring).

 Although the EFPIA/ABPI Codes have different rules for reporting* prospective* (R&D payments) and *retrospective* (non-R&D payments) non-interventional studies, they were often listed jointly, forming the second largest category of “non-interventional studies” (15%). Instances in which the two study types could not be distinguished were sometimes approached differently. For instance, Bristol Myers Squibb and Novartis reported them all as non-R&D payments (potentially on a name basis), while MSD – as R&D payments (in aggregate by default). Astellas, also listed, without an explanation, “non-interventional studies that are *retrospective* in nature” (emphasis added) in the R&D category, although it should have been disclosed on a name basis as non-R&D payments following the EFPIA Code.^78^

 Some companies included other forms of “research,” including “basic research,” “research collaborations” (12%), and “real world data studies and Health Outcomes research” (8%). R&D was also described as referring to *all* studies underpinning regulatory submissions, such as applications for marketing authorisation, health technology assessment or drug reimbursement (4%).

 Some companies specified which study types were disclosed as non-R&D payments, including, consistent with the ABPI Code, market research involving participants known to the companies (26%), and research *not* intended for regulatory submissions (6%).

 Some payments were excluded from disclosure altogether, including anonymous market research (17%). Further, although payments made to healthcare professionals or organisations *indirectly* via CROs were disclosed as R&D payments (32%), payments made *directly* to CROs for their services were not reported (15%). Payments for study drugs were sometimes reported differently. For example, while Sobi and Pharma Mar included them as R&D payments, Chugai and Roche excluded them from disclosure. Similarly, although Pharma Mar listed administrative costs as R&D payments, Grünethal and Brinannia did not disclose them.

 Disclosure requirements are part of the ABPI Code, and therefore oversight and sanctions follow the same principle as with any other content of the Code.^84^ Specifically, it is the PMCPA – the ABPI’s self-regulatory body – that retrospectively investigates potential breaches when these are brought to its attention through complaints. Consistent with the idea that the PMCPA also retrospectively monitors disclosure requirements, it lists nine cases between 2016-2019 that involved possible breaches of disclosure requirements. In six of the nine cases, companies were found to be in breach, but only one appeared to involve R&D payments – in 2017, A Menarini voluntarily admitted to have missed timely disclosure of an R&D payment to a UK organisation, allegedly because of internal miscommunication.^125^ Overall, the reviewed cases suggest that although the PMCPA does monitor compliance with disclosure obligations, there is no evidence it has concerned itself with the issue of how companies actually define R&D.

###  Scope of Disclosure – Public Regulation

 In all but one country with mandatory disclosure, R&D payments are disclosed publicly; in Turkey, however, all payments are only disclosed to state authorities (Table 3). Also, except Turkey, where the legislation does not introduce specific payment categories, R&D payments are disclosed under other payments. This approach contrasts with the EFPIA Code, featuring R&D as one of the main payment categories. Further, activities associated with R&D are described in detail ranging from specific study types (Slovakia) to “research” (Denmark). All countries provide an exhaustive (full) list of R&D activities except Portugal, where “clinical studies” or “trials” are only indicated as examples.

**Table 3:** Scope of Disclosure of R&D Payments in Countries With Public Regulation

**Country**	**France**	**Portugal**	**Turkey**	**Denmark**	**Slovakia**
Name of regulation^a^	Law No. 2011-2012 (Law Bertrand)	Decree Law 20/2013 and 128/2013	Regulation on Promotional Activities of Medicinal Products for Human Use 2015	Health Act of 2014, Executive Order No. 1153	Act No. 362/2011 on Medicines and Medical Devices
Overseeing authority	Ministry of Social Affairs and Health	National Authority of Medicines and Health Products	Ministry of Health	Danish Medicines Agency	National Health Information Center
**Provisions Affecting the Scope of Disclosure Directly**					
Categorisation of R&D payments	Disclosed within three major payment categories – advantages (benefits); contracts (agreements); payments related to contracts (remuneration)	Fall within other payment categories, including “subsidies, sponsorships, grants or any other value, good or right granted or received”	No list of disclosed payment categories is provided	One of disclosed “professional affiliations” with industry	Falls under “financial and in-kind benefits”
Activities associated with R&D payments	Scientific research; consultancy contracts with scientific researchers; contracts regarding scientific expertise; research; clinical study of a biological material	Clinical studies and trials	N/A	Research	Clinical trials, non-intervention clinical studies, post-registration studies of human drug safety, market research
List of activities associated with R&D payments	Exhaustive	Indicative	N/A	Exhaustive	Exhaustive
**Provisions Affecting the Scope of Disclosure Indirectly**					
Funders	Pharma, medical device, in vitro diagnostic medical device, cosmetics, veterinary products, tattoo products, associated providers	Pharma, medical device, other (unspecified)	Pharma	Pharma, medical device, and specialty stores with medical equipment	Pharma
Recipients	Individual and organisational	Individual and organisational	Individual and organisational	Individual	Individual and organisational (only healthcare providers)
Exempted payments	Below €10 including taxes	Below €60	Below 10% of the minimum monthly salary	No	No

Abbreviations: N/A, not available; R&D, research and development. ^a^The dates provided in this row refer to when public regulation of payment disclosure was first introduced. If public regulation of payment disclosure forms part of a larger piece of legislation, we specify, where appropriate, if the regulation of payment disclosure was introduced as a change to already existing legislation. The dates reported here do not cover changes to, or refinements of, legislative provisions focusing on payment disclosure.

 The scope of disclosed R&D payments is at least equal to the provisions of the EFPIA Code in all countries bar Slovakia. As the Portuguese and Turkish legislation mandates the disclosure of *all* payments, the R&D payments covered by the EFPIA Code would, ipso facto, be included. The same should be achieved via the Danish legislation, which has a broad (and unspecified) category of “research”; and the French legislation, covering five distinct categories of research activities, including consultancies and contracts. Contrastingly, in Slovakia, only two of the study types covered by the EFPIA Code are disclosed, with payments related to non-clinical (laboratory) studies not listed as subjected to disclosure.

 In addition to the measures referring to R&D payments *directly*, the scope of disclosure is *indirectly* affected by industry funders covered by the legislation, with the Danish and Portuguese provisions also including medical device manufacturers, and the French – multiple healthcare relevant industries. However, the scope of disclosed R&D payments is constrained in the Slovak legislation, which mentions the disclosure of R&D payments by companies being *marketing authorisation holders*, with R&D payments made by other funders falling under self-regulation.^105^ Likewise, the scope of disclosure is *indirectly* shaped by recipients mentioned in disclosure requirements. In all countries, disclosure covers payments to both healthcare professionals and organisations except for Denmark, where only healthcare professionals are covered.^121^ In addition, in Slovakia, R&D payments to *non-healthcare providers*, such as “universities and scientific institutions,” are disclosed under the EFPIA Code.^105^ The final set of indirect influences relates to payments falling below a certain value being excluded from disclosure in Denmark, France, and Turkey.

###  Country-Level Payment Distribution 

 We were able calculate the share of R&D payments within the overall industry payments in 14 countries disclosing R&D payments following exclusively the EFPIA Code in 2019 (Table 4).

**Table 4:** Country-Level Distribution of R&D Payments in 14 European Countries With Self-regulation of R&D Payment Disclosure (2019)

**Country**^a,b,c,d^	**Disclosed R&D payments**^e^	
	**€m (% All Payments**^f^**)**	**€m Per m Inhabitants**^g^
UK	435 (70%)	7
Germany	404 (64%)	5
Spain	259 (43%)	6
Belgium	143 (64%)	12
The Netherlands^h^	135 (68%)	8
Poland	106 (64%)	3
Sweden	73 (82%)	7
Switzerland	62 (37%)	7
Austria	59 (44%)	7
Finland	25 (63%)	5
Ireland	21 (59%)	4
Romania^i^	20 (42%)	1
Norway	13 (68%)	2
Czech Republic	N/A (70%)	N/A
**Total**	**1766**	**5**

Abbreviations: N/A, not available; R&D, research and development. ^a^Data sources:
Austria,^126^ Germany,^127^ Poland,^128^ Spain,^129^ the Netherlands,^130^ and Switzerland^131^ – publicly available national pharmaceutical industry group press releases. Belgium, Ireland, Romania, and the UK – eurosfordocs.eu. Czech Republic – the EFPIA 2019 Europe-wide summary report.^81^ Finland,^132^ Norway,^133^ Sweden,^134^ – industry trade groups’ response to the stakeholder survey. Romania – a national trade group report^93^ and individual drug company websites signposted in the report (R&D payments), and eurosfordocs.eu (non-R&D payments). When more than one data point existed for a country the data sources were prioritised as follows: information obtained directly from pharmaceutical industry trade groups or public or public-private authorities; information published by national pharmaceutical industry trade groups or extracted from eurosfordocs.eu; the 2019 EFPIA Europe-wide report collating data from its member trade groups.^81^ ^b^ In eurosfordocs.eu, which provided payment data for Belgium, Ireland, Romania, and the UK, payment values in non-euro currencies were converted to euros using CurrencyConverter,^135^ a Python library for exchange rates. In all other instances, we used average yearly exchanged rates published by the European Central Bank.^136^ ^c^ Countries are sorted descendingly based on the value of R&D payments. ^d^ The statistics for Belgium and the Netherlands cover both the pharmaceutical and medical devices industries. The statistics for other countries cover the pharmaceutical industry only. ^e^ In countries adopting self-regulation based on EFPIA’s definition of R&D payments, these payments should be related to non-clinical studies, clinical trials, and prospective non-intervention studies.^75^ ^f^ All disclosed payments include R&D and non-R&D payments. ^g^ The population-level data was obtained from a Eurostat news release. The data is reported as of 1^st^ January 2019 in all countries except for Norway, where it is reported as of January 1, 2018.^137^ ^h^ Non-R&D payments reported in the Netherlands do not follow the categories from the EFPIA Code. However, R&D payments are reported consistent with the EFPIA Code, which includes the lack of disclosure on a named basis. This means that R&D payments reported in the Netherlands can be added to R&D payments reported in the other countries. The sum of non-R&D payments reported in the Netherlands was obtained by adding payments made to healthcare professionals or organisations. Therefore, the sum of payments reported in the Netherlands can be compared with the other country sums from the table. ^i^ The exact figures for Romania include €28m (calculated based on data reported in a publicly-run disclosure platform) and €20m of R&D payments (calculated using a pharmaceutical industry trade group report and data from individual drug company websites – see footnote a).

 The value of R&D payments ranged widely, with companies making in absolute terms over 33 times more such payments in the United Kingdom than Romania. A similar contrast in the overall value of R&D payments existed between Germany and Norway. Country differences were less pronounced when considering population size, with a 12-fold difference between Belgium and Romania.

 Across the 14 studied countries, R&D payments constituted almost 60% of all payments (€1.8bn of €3.0bn). In 10 of these countries, R&D payments represented over half of the value of all payments. In addition, in 7 countries with available breakdown of non-R&D payments, R&D payments were the single largest payment category (Table S1, Supplementary file 2).

 We also collected data for France and Slovakia, where legislation introduced unique payment categories (Table 5). The value of R&D payments reported in France was lower than in Germany and the United Kingdom, but the difference was less pronounced considering the population size. The value of R&D payments in Slovakia was the lowest, both in absolute terms and accounting for population size. The share of R&D payments was also the lowest, indicating that a significant share of R&D might be disclosed under self-regulation, including those made to research institutions, such as universities.

**Table 5:** Country-Level Distribution of R&D Payments in Two European Countries With Public Regulation of R&D Payment Disclosure (2019)

**Country**^a,b^	**Breakdown of Disclosed R&D Payments**^c^		**All Disclosed R&D Payments**^d^	
	**Study Types **	**€m (% All R&D Payments**^e^**)**	**€m (% All Payments)**	**€m Per m Inhabitants**^f^
France^g^	Scientific research	252 (66%)	383 (43%)	6
	Consultancy contracts with scientific researchers	108 (28%)		
	Contracts regarding scientific expertise	22 (6%)		
	Research	1 (0%)		
	Clinical study of a biological material	0 (0%)		
Slovakia^h^	Clinical trials	6 (100%)	6 (19%)	1
	Post-authorisation studies of drug safety	0 (0%)		
	Non-interventional clinical studies	0 (0%)		

Abbreviation: R&D, research and development. ^a^Data sources:
France – eurosfordocs.fr, an independent online platform which enhances the accessibility of data payment data disclosed in the public database Transparence Santé. Slovakia – payment database available from the website of Národné Centrum Zdravotníckych Informácií.^122^
^b^ The French data covers payments made by pharmaceutical companies (manufacturers of “human drugs”). Payments reported by Slovakia also cover payments made by pharmaceutical companies. ^c^ The breakdown of R&D payments presented in the table follows the study types reported in the French and Slovak datasets. ^d^ All disclosed R&D payments are the sum of specific types of R&D payments. ^e^ All payments include R&D and non-R&D payments. ^f^ The population-level data was obtained from a Eurostat news release. The data is reported as of January 1, 2019.^137^ ^g^ The exact figures for France are as follows: €252 397 511 (scientific research); €107 958 348 (consultancy contracts with scientific researchers); €21 962 381 (contracts regarding scientific expertise); €531 681 (research); €0 (clinical study of a biological material). ^h^ The exact figures for Slovakia are as follows: €6 230 311 (clinical trials), €34 659 (non-interventional clinical studies), and €178 823 (post-authorisation safety studies of a medicinal product for human use).

###  Company-Level Payment Distribution

 We collected complete company-level data from 2017 to 2019 for three countries disclosing R&D payments following the EFPIA Code – Belgium, Ireland, and the UK (Table 6).

**Table 6:** Company-Level Distribution of All Payments and R&D Payments in Belgium, Ireland, and the United Kingdom (2017-2019)

	**UK**			**Belgium**			**Ireland**		
	**R&D Payments (€)**	**All Payments (€)**	**R&D Payments as % All Payments**	**R&D Payments (€)**	**All Payments (€)**	**% R&D Payments**	**R&D Payments (€)**	**All Payments (€)**	**% R&D Payments**
Median (IQR)	295 089 (0–3 188 268)	1 338 212 (210 583–7 233 079)	32.8% (0%–68%)	0% (0%–0%)	60 448 (13 205–342 131)	0% (0%–0%)	195 861 (0 –1 482 405)	953 706 (261 021 –2 453 010)	27% (0%–47%)
Maximum	167 143 325	183 036 733	100%	55 238 184	69 381 760	98%	15 877 914	15 951 700	100%
Gini Index	0.86	0.82		0.96	0.91		0.76	0.63	
Top 10 donor share	66%	60%		71%	55%		82%	70%	
Top 20 donor share	86%	81%		90%	74%		97%	89%	
Top 10 donor share within global Top 20 largest companies	100%	100%		90%	90%		90%	90%	
Top 20 donor share within global Top 20 largest companies	80%	85%		75%	75%		75%	75%	

Abbreviations: R&D, Research and Development; IQR, interquartile range. *Notes. *The R&D payment data reported in this table was extracted from eurosfordocs.eu.^86^ The company size data (the top 20 companies) was calculated using three combined yearly rankings of the top 50 largest companies based on global sales values published Pharmaceutical Executive.^96-98^ The years covered by the sales rankings corresponded with the years in which the payment disclosures were made.

 In each country, a few funders, typically big pharma companies, provided most R&D payments (Table 6; see also Tables S2-S4 of Supplementary file 2). These funders were highly similar across the countries, with a 75%-90% overlap between the top 20 lists (Table S5, Supplementary file 2). The concentration of R&D payments was the highest in Belgium, as demonstrated by the interquartile range values and Gini indexes (Table 6). This most likely reflects the fact that the Belgian payments database includes both pharmaceutical and medical device manufacturers, and has the largest number of companies (491 versus 140 and 45 in the United Kingdom and Ireland, respectively), many of which made only small payments. For example, the share of companies making R&D payments over €1 000 000 during the three-year period was 7% in Belgium (34 of 491), compared to 38% (53 of 140) in the United Kingdom and 27% (12 of 45) in Ireland. Nevertheless, the concentration by major funders was the highest in Ireland, as demonstrated by the shares of R&D payments held by the top 10% or 20% companies (Table 6). Notably, the largest funder, Allergan (subsequently merged with AbbVie), made payments representing 30% of all R&D payments in Ireland.

 Consistent with the pattern of payment concentration, the share of companies with a limited focus on R&D payments was the highest in Belgium, as shown by the percentage interquartile range values. Nevertheless, the share of companies with R&D constituting over 90% of their payments was the highest in the United kingdom – 7% (10 of 140), contrasted with 2% in Ireland (1 of 45) and 1% in Belgium (5 of 491).

 Each country had a largely unique composition of companies with the highest R&D payment shares, with the shared ones between 30%-35% (Table S5). In Belgium and the United kingdom, many companies with a near-exclusive focus on R&D payments were typically small, as measured by their revenues, with narrow drug portfolios oriented around rare disorders. As these companies often concentrate on developing several key products, their payments were unsurprisingly predominantly associated with R&D.

 In the United Kingdom, the highest R&D payment share (100%) was held by Clinuvel (Table S4), a biotechnology company which for over a decade has prioritised developing and marketing a single drug for a rare metabolic skin disorder.^138^ Other examples include Biotest (R&D = 98% of all payments), a firm with the portfolio including two drugs for rare bleeding disorders, and a few clinical trials in recent years.^139^ Yet another one is Bluebird Bio (R&D = 97% of all payments), a company concentrating on rare genetic disorders, including a gene therapy for an acute form of transfusion-dependent β-thalassaemia.^140^ In Belgium, Oxurion (formerly Thrombogenics) had the highest R&D payment share (R&D = 98% of all payments), a company with a single recently launched drug for a retinal vascular disorder.^141^ Another one is Sarepta (R&D = 97% of all payments), a company concentrating exclusively on four rare genetic diseases.^142^ Contrastingly, big pharma was dominant among companies with the highest R&D payment shares in Ireland. One exception was Sobi (R&D = 73% of all payments), a company prioritising rare diseases in haematology and immunology.^143^

## Discussion

 Criticisms of self-regulation of drug company payment disclosure in European countries typically emphasise high levels of non-disclosure by healthcare professionals receiving non-R&D payments.^78,79,87,144,145^ However, we suggest that R&D payments are an even greater challenge to transparency of financial relationships between the pharmaceutical industry and the healthcare sector.

 Although EFPIA commits, at least rhetorically, to increasing individual consent rates for non-R&D payments,^146,147^ it allows, by default, the recipients of R&D payments to remain undisclosed. Paradoxically, then, non-R&D payments whose recipients refused to be named are *more* transparent than R&D payments because they at least distinguish between payments to individuals and organisations.^145,148^

 Trade group and company disclosure practices may further erode EFPIA’s minimum standards. Our study of companies participating in Disclosure UK indicates that while they typically agree on the core R&D study types, some extend or even contravene the EFPIA definition, without evidence of this prompting inquiries and penalties from the industry self-regulatory body.

 The disclosure practices associated with self-regulation contrast with the US Sunshine Act as well as Danish, French, and Portuguese legislation, which, by covering *all* research activities, removes ambiguity in interpreting which study types ought to be reported. Further, under self-regulation, companies are largely free to decide which activities they consider as “related to” or “essential for” R&D. Contrastingly, under the US Sunshine Act, for example, *all* consultancies, and travel and lodging support are non-research payments. The French and Slovak cases further demonstrate the legal and technical feasibility of separating non-R&D and R&D payments in the European context.

 Self-regulation likely results in overreporting of R&D payments and, consequently, underreporting of non-R&D payments. Indeed, of the 14 studied countries with self-regulation, 11 had R&D shares higher than France, a country with comprehensive R&D and non-R&D payment disclosure. Putting aside the possibility of intentional hiding of certain payments within the R&D category, some companies may genuinely interpret what they see as “essential” for R&D more expansively. Companies also lack incentives to report costs “subsidiary” to R&D on a name basis. As disclosed payment data informs corporate intelligence gathering,^149^ reporting subsidiary costs as non-R&D payments could bolster competitors’ commercialisation and marketing strategies. Further, large R&D payments serve to demonstrate commitment to creating innovative medicines and healthcare investment, while also helping justify higher drug prices and regulatory incentives for pharmaceutical R&D.^18,20,27^

 The confusion surrounding how the company shares of R&D payments are calculated reflects controversies regarding some of the industry-endorsed estimates of pharmaceutical R&D.^13,21,150^ The two problems are interconnected as R&D payments are part of R&D spending, covering, for example, in-house R&D and all costs of conducting clinical trials. Nevertheless, as far as we know, EFPIA does not clarify how R&D payments relate to R&D spending.^151^ Similarly, company methodological notes typically provide little additional insight.

 Moreover, the high shares of R&D payments – absent information about their recipients – may prevent scrutiny of key COIs, including payments for clinical trials which be vital for co-opting Key Opinion Leaders to influence the medical opinion and policy-makers.^28,152-156^ Evidence also exists of some clinical trialists failing to disclose some COIs with the industry.^157,158^ These COIs may be associated with payments made by top big pharma funders or small biotech firms reporting (nearly) all payments as R&D while seeking approval and public funding for exceedingly expensive therapies, such as Bluebird Bio’s Zynteglo, branded “the second most costly treatment in history.”^159^

 Against this background, EFPIA recognises the need to “[e]xplain R&D figures and why they are disclosed in aggregate.”^77^ Aggregate disclosure corresponds with the view, expressed by EFPIA’s Director-General, that “Some relations are no ‘conflicts’ [of interest], such as clinical research.”^160^ Aggregate disclosure is also presented as consistent with a self-regulatory approach designed to focus on payments for meetings and services, while the disclosure of information on industry-sponsored clinical trials is ensured by the EU Clinical Trials Directive (2001/20/EC), requiring drug trials registration and public summary reporting, and the European Medicines Agency (EMA) Transparency Policy (Policy 0070), providing public access to clinical study reports submitted by companies to the EMA.^161^ Nevertheless, some, often small- to medium-size, companies’ compliance with key requirements of the Clinical Trials Directive has been poor,^162^ and the impact of a new comprehensive Clinical Trials Information System, scheduled for 2022, remains to be seen.^163^ The EU has also been expanding transparency and COI policies relevant for laboratory (Regulation 2019/1381) and registry-based studies (EMA’s Guideline on Registry-based studies).

 The industry further defends its approach to R&D payment disclosure by stressing potential reporting problems caused by international R&D activities, particularly multi-centre clinical trials.^164^ However, methodological notes suggest that companies successfully ascertain the ultimate payment recipients using the primary place of residence (eg, Shionogi and Pharma Mar in the United Kingdom^124^). Further, disclosure to named recipients is allegedly incompatible with the “commercial sensitivity” of R&D payments,^124^ which is protected by competition law.^165^ This argument is challenged by the Australian evidence industry trade group supporting mandatory disclosure of consultancy payments related to R&D.^44^

 Only five of the 37 studied European countries have sought to address the concerns about the commercial sensitivity of R&D payments by making their disclosure compulsory. Nevertheless, public regulation can be suboptimal, as demonstrated by the cases of Spain, Belgium, and the Netherlands, where the mandatory disclosure of non-R&D payments has not been extended to R&D payments. Further, even in countries mandating disclosure of R&D payments, such as Slovakia, its extent can be diminished indirectly by the scope of funders or recipients falling under disclosure requirements.^43,44^

 Data presentation is another challenge.^43,76^ Only in France and Slovakia are R&D payments itemised and available for analysis, although in France it is thanks to an independent disclosure platform, eurosfordocs.fr, offering user-friendly access. Contrastingly, identifying R&D payments in the Danish and Portuguese databases would require scrutinising each payment description. Finally, only in three countries can payment data be connected to other databases – in Denmark and France via recipient identifiers, while in Slovakia – via clinical trial numbers. Limited data interconnectedness is a key obstacle in studying companies’ marketing strategies as this requires, among others, information on prescriptions.^76,166^ Similarly, a key way to understanding the nature and extent of COIs and estimating their effects on research^36,37,40^ is via linking information on studies, investigators, and study sites to company payment data detailing the value, forms and role of R&D funding.

###  Limitations

 Our study has limitations. As the methodological notes lack a single reporting format, the full spectrum of disclosure practices of Disclosure UK participants remains unknown. For example, companies providing less detail might have made fewer R&D payments and therefore had little to comment on; alternatively, they might not have wished to specify what was disclosed. Conversely, companies presenting extensive lists of R&D payments might have been more diligent and transparent than those mentioning fewer disclosed R&D payments.

 In countries with public regulation, practical challenges in disclosing R&D payments might have been concealed by the lack of technical guidance or company equivalents of “methodological notes” used under self-regulation. Further, we assumed that companies fully followed the legal provisions, which may not always be true. Overall, we may have overstated the advantages of public regulation over self-regulation.

 We could not collect R&D payments from 17 of 32 countries with self-regulation lacking centralised databases. Nevertheless, these countries are unlikely to have payment patterns radically different to those we identified, as demonstrated by cross-country analyses of non-R&D payments.^79^ Further, as France and Slovakia had considerably different R&D payment shares, similar differences might exist between the three other countries with public regulation where we were unable to collect R&D payment data.

## Conclusions and Policy Recommendations

 One short-term improvement to the transparency of R&D payment reporting under the EFPIA Code would involve breaking down R&D payments into those associated with each of the three core study types and distinguishing between organisational and individual recipients. Some companies have already done this voluntarily in the United Kingdom (eg, Syner Med). The reporting of activities associated with each study type should be standardised (eg, following the example of Mitsubishi Tanabe) and distinguished from non-R&D payments, including detailed guidance on which costs may count as subsidiary to R&D.

 Nevertheless, the public interest in full transparency of R&D payments over demands for protecting commercial secrecy^32^ can be most effectively secured via a new EU regulation or directive mandating disclosure using clear definitions of research activities and payment types. The payments should be disclosed in a centralised database with an accessible interface, allowing for integration with existing and planned EU databases, via funder, recipient, and activity (eg, clinical trial) identifiers.^43,76^ A centralised disclosure system would match the cross-European nature of today’s clinical research, for example, multi-country trials.^167^ By providing some financial details about trials, including potential COIs, it would complement on-going initiatives seeking to enhance the transparency of clinical trial data reporting, including details of the roles of study sponsors.^168-170^ Given the evidence of the impact of pharmaceutical lobbying at the EU^171^ and national levels^153,172^ the legislative process must itself be transparent and involve broad public consultation.

 Further, R&D payments should be incorporated into existing national disclosure systems, including compulsory registers of interests reported by physicians,^173,174^ authors of treatment guidelines, or members of advisory committees,^175^ such as those evaluating technologies applying for public funding.^176,177^

 Enhancing the transparency of R&D payment data alone is unlikely to make funders or recipients more accountable.^178,179^ Notably, the disclosure of payments under the US Sunshine Act has not eliminated instances of corrupt relationships between companies and physicians^180,181^ or reduced physicians’ acceptance of COIs.^182^ The limited extent of behavioural change may be caused by the absence of pressure from the public, which, in turn, results from its low engagement with payment data.^183^ One way of addressing this challenge is via supporting the activity of transparency watchdogs or data platforms, such as eurosfordocs.fr, which have triggered highly publicised investigations into COIs.^184-186^

## Acknowledgements

 We would like to thank all pharmaceutical industry trade groups and public authorities which have responded to our request for information and took the time to explain the nature of local regulatory solutions. We are especially grateful to the ABPI’s Disclosure UK Team for their continued interest in our research and openness in sharing details of their work. We also thank colleagues who commented on drafts of this paper and offered linguistic assistance: Marcell Csanadi, Eniko Csanadi, Alice Fabbri, Kevin Jean, Fatma Korkmaz, Olga Loblova, Savvas Morris, Emily Rickard, Eszter Saghy, Christos Vasilakis, and Weronika Ozieranska

## Ethical issues

 This study did not require a full ethics approval as it relied on publicly available data aggregated at the country or organizational level. The study’s ethical implications were approved via a peer ethics review process at the Department of Social and Policy Sciences, University of Bath in February 2020.

## Competing interests

 PO’s PhD student was supported by a grant from Sigma Pharmaceuticals, a UK pharmacy wholesaler and distributor (not a pharmaceutical company). The PhD work funded by Sigma Pharmaceuticals is unrelated to the subject of this paper. LM and PAJ are members of Euros for Docs, a non-profit organization registered in France that seeks to promote transparency of drug company funding in the healthcare sector by making payment data accessible and complete across Europe. PAJ is employed by Haute Autorité de Santé, the French independent health technology assessment organisation. SM’s partner is employed by PRA Health Sciences, a global Contract Research Organization whose costumers include many pharmaceutical companies.

## Authors’ contributions

 PO conceived and wrote the paper, collected, and analysed the data. LM created the eurosfordocs.eu database, collected the data, and contributed to writing. PAJ collaborated with LM on creating eurosfordocs.eu, collected the data, and contributed to writing. SM conceptualized the paper and contributed to writing.

## Funding

 This work (SM as Principal Investigator and PO as Co-Investigator) was supported by the grant ‘Following the money: cross-national study of pharmaceutical industry payments to medical associations and patient organisations,’ awarded by The Swedish Research Council (VR), no. 2020-01822.

## Supplementary files

Supplementary file 1. Methods.

Supplementary file 2. Background Data.^81,93-95,126-134,136,187-190^
