# Supplementary material for: Tip of the Iceberg? Country- and Company-Level Analysis of Drug Company Payments for Research and Development in Europe
Source: Int J Health Policy Manag. 2022 Mar 15;11(12):2842–59. doi: 10.34172/ijhpm.2022.6575 (PMC10105170; doi:10.34172/ijhpm.2022.6575)
Supplement: Supplementary file 1 — Methods. [file ijhpm-11-2842-s001.pdf]

Article title: Tip of the Iceberg? Country- and Company-Level Analysis of Drug Company Payments for Research and Development in Europe

Journal name: International Journal of Health Policy and Management (IJHPM)

Authors' information: Piotr Ozieranski[1]*, Luc Martinon[2], Pierre-Alain Jachiet[2], Shai Mulinari[3]

[1]Department of Social and Policy Sciences, University of Bath, Bath, UK.

[2]Euros for Docs, Paris, France.

[3]Department of Sociology, Lund University, Lund, Sweden.

(*Corresponding authors: p.ozieranski@bath.ac.uk)

Supplementary file 1. Methods

Stakeholder survey, Part 1: Questions to national pharmaceutical industry trade groups

Dear Sir or Madam

I would be grateful if you could answer a few questions listed below regarding the way in which your organisation and its Members disclose transfers of value to healthcare professionals and organisations as well as patient organisations. Your answers would be very helpful in informing scholarly publications on which I am currently working that are seeking to present a full and accurate picture of transfers of value in Europe. Please let me know should you have any questions about this research.

[if relevant] In particular, your answers would help me expand on the information your Association provided in a recent EFPIA report and on its webpages, including here. Some of the matters covered in my questions have been touched upon in the sources of data referred to above, although incompletely. I would therefore be grateful if you could answer my questions so that I report correct and accurate information in any research outputs.

Thank you very much in anticipation for your valuable time in answering my questions.

I look forward to hearing from you.

Sincerely yours

[anonymised]

I would be grateful if you could answer the following questions regarding your Association

1. What is the number of member companies in your Association?
2. How many companies which are not members of your Association follow its Code of Practice?
3. Please could you send me the most recent version of your Association's code of practice (if available, I would appreciate an English version of the document)?

Please could you answer the following questions regarding the platform and mechanisms of disclosure of transfers of value.

4. What is the platform for disclosing transfers of value to healthcare professionals and organisations (e.g. individual pharmaceutical company websites, a single database of transfers of value from all companies)?
5. If transfers of value are disclosed on individual pharmaceutical company websites, does your Association have a website providing links to these websites? If so, please could you provide me with a web link?
6. If transfers of value are disclosed on individual pharmaceutical company websites, has your Association considered creating a single database of transfers of value from all companies?
7. Do healthcare professionals in your country have to consent to the transfers of value being publicly disclosed; or is the disclosure of transfers of value to healthcare professionals mandatory (i.e. healthcare professionals are not asked to consent)? If the disclosure of transfers of value they received is mandatory, please could you state the regulation which makes them mandatory?
8. If healthcare professionals in your country have to consent to the transfers of value being publicly disclosed, does your Association have any expectations regarding the minimum HCP consent rate that should be achieved by companies signing up to its Code of Practice? If so, please could you specify this consent rate? Further, does your Association request companies achieving lower-than-expected healthcare professional consent rates to explain why this might be the case and identify possible ways of improving the consent rates?
9. If healthcare professionals in your country have to consent to the transfers of value being publicly disclosed, has your association considered implementing a simple summary disclosure rate statistic that is easily comparable between companies, such as the percent of the amount of transfers of value disclosed in the aggregate and/or the percent of healthcare professionals disclosed in the aggregate?
10. If healthcare professionals in your country have to consent to the transfers of value being publicly disclosed, does your association have any specific guidelines on how HCP disclosure rates could be improved by its Member Companies? If so, I would be grateful if you could share these guidelines with me?
11. If healthcare organisations have to consent to the transfers of value being publicly disclosed could you provide examples of reasons that would necessitate placing them in the aggregate disclosure?
12. Please could you state the threshold for the acceptable value of meals and drinks that was set by your association?
13. If some transfers of value need to be disclosed in a database run by a public institution, are there any transfers of value (e.g. research and development) that are still disclosed by pharmaceutical companies themselves. If so, I would be grateful if you could specify what these transfers of value are and why they are disclosed by pharmaceutical companies.
14. As far as your Association is aware, do companies disclosing their transfers of value use "unique country identifiers" recommended in the EFPIA Code of Practice, and not only recipient names or locations, to distinguish payment recipients? If so, are these identifiers shared among different companies so that they can identify the same recipient using the same identifier?

15. Did members of your Association (or other companies following your Association's code of practice) disclose transfers of value to healthcare professionals and organisations in 2020 (i.e. covering payments made in the 2019 calendar year)?

If members of your Association (or other companies following your organisation's code of practice) disclosed transfers of value to healthcare professionals and organisations in 2020 (i.e. covering the 2019 calendar year) please could you answer the following questions.

16. How many pharmaceutical companies in total disclosed transfers of value to healthcare professionals and organisations made in 2019? How many of those companies were members of your Association?
17. Is the number of companies which did not disclose any transfers of value made in 2019 known? If so, please could you state this number? How many of those companies were members of your Association?
18. What was the overall share of healthcare professionals consenting to their transfers of value being disclosed in 2019?
19. What was the overall share of healthcare organisations consenting to their transfers of value being disclosed in 2019?
20. What was the total value of all non-research transfers of value to all healthcare professionals and organisations made in 2019?
21. What was the total value of all non-research transfers of value to all healthcare organisations made in 2019?
22. What was the value of all research-related (i.e. R&D) transfers of value to healthcare professionals or organisations made in 2019?
23. What share of the overall value of transfers of value made to healthcare professionals disclosed at the individual level in your country in 2019?
24. What share of the overall value of transfers of value made to healthcare organisations was disclosed at the individual level in your country in 2019?
25. What was the value of grants and donations made to healthcare organisations in 2019?
26. What was the value of fees for services and consultancy made to healthcare organisations in 2019?
27. What was the value of contributions to costs of events made to healthcare organisations in 2019?
28. What was the value of fees for services and consultancy made to healthcare professionals in 2019?
29. What was the value of contributions to costs of events made to healthcare professionals in 2019?

Finally, I would be grateful if you could answer the following questions regarding the public disclosure of transfers of value to patient organisations.

30. Please could you send me the most recent version of your Association's code of practice as it relates to working with patient organisations (if available, I would appreciate an English version of the document)?
31. Are there any companies that are not members of your Association which disclose transfers of value to patient organisations in line with the EFPIA Code of practice?
32. How many companies disclosed transfers of value to patient organisations made in 2019? How many of those companies were members of your Association?
33. What was the total value of all transfers of value to patient organisations made in 2019?

Stakeholder survey, Part 2. Questions to public or multistakeholder bodies overseeing pharmaceutical industry payment disclosure

Dear Sir or Madam

I would be grateful if you could answer a few questions listed below regarding the way in which your institution discloses payments made by drug companies to individuals and organisations within the healthcare system in your country. Your answers would be very helpful in informing scholarly publications on which I am currently working that are seeking to present a full and accurate picture of payments made by the pharmaceutical industry in Europe. Please let me know should you have any questions about this research.

Some of the matters covered in my questions have been touched upon on your website. However, I would be extremely grateful for your answers to avoid any misunderstandings which may be caused by language issues, particularly as they relate to complex regulatory matters.

Thank you very much in anticipation for your valuable time in answering my questions.

I look forward to hearing from you.

Sincerely yours

[anonymised]

I would be grateful if you could answer the following questions regarding the nature of payments disclosed by [insert name of institution]

1. What industries are covered by the disclosure requirements (e.g. pharmaceutical, medical device, veterinary)?
2. Please could you briefly describe what is meant by each type of the industry covered by the disclosure?
3. What types of payment recipients are covered by the disclosure requirements (e.g. healthcare professionals, healthcare organisations, patient organisations)? Are these definitions consistent with the ones used in the EFPIA Code of Practice (https://www.efpia.eu/media/554677/efpia-code-2020.pdf) ?
4. Please could you briefly describe what is meant by each type of recipient (e.g. what is meant by the healthcare processional or healthcare organisation)? Are these definitions consistent with the ones used in the EFPIA Code of Practice?
5. What are the categories of payments covered by the disclosure managed by your institution (e.g. sponsorship of conference attendance, consultancy fees, research and development)? Are they the same one as those used in the EFPIA Code of Practice?
6. Please could you briefly describe what is meant by each type of payment (e.g. what is meant sponsorship of by conference attendance)? Are these definitions consistent with the ones used in the EFPIA Code of Practice?
7. If the definitions of payments or recipients used by your institution are not consistent with the ones used in the EFPIA Code of Practice, please could you explain why this is the case? In other words, why alternative definitions have been created for the reporting purposes of your institution as opposed to using the ones introduced by the EFPIA Code of Practice?
8. Are all payments to all payment recipients publicly disclosed by your organisations or are any payments or recipient types exempted from public disclosure?
9. Are payments reported by your institution reported on an individual basis (i.e. each payment has a separate database entry) or are they aggregated on a yearly basis (i.e. all payments of a certain type from a certain company are reported jointly)?
10. What are the responsibilities of companies making payments in relation to their disclosure?
11. What are the responsibilities of payment recipients in relation to their disclosure (e.g. do they need to disclose payments themselves or just verify disclosures made by companies making payments)?
12. Are payment disclosures managed by your institution reported based on calendar years or financial years? If payments are made based on financial years, please could you specify what are the start and end dates of a financial year in your country?
13. Are payment values made publicly available by your institution the same as those received by payment recipients (e.g. do they include the value of any taxes, such as VAT, paid by companies making the payments)?
14. If payments reported in by your institution are not reported consistently with or without relevant taxes (e.g. VAT), where might database users find information on approaches to tax reporting taken by each company making payments.
15. Do all industries covered by the payment disclosures managed by your institution report payments in exactly the same way (e.g. using the same definitions of payments, payment categories and recipients)?
16. Is the database of payments managed by your institution downloadable or not? Please could you explain why the decision has been made to make it downloadable (or not)?
17. Please could you send me the most recent copy of the regulation and/or policy which governs the disclosure of payments made your institution (if available, I would appreciate an English version of the document)?

Please could you answer the following questions regarding the disclosure requirements covered by your institution and the self-regulatory disclosure system overseen by the European Federation of Pharmaceutical Industries (EFPIA), as described in its Code of Practice?

18. Do pharmaceutical companies in your country have to disclose payments with your institution as well as in line with the disclosure system managed by EFPIA (i.e. do companies need to disclose payments to healthcare professionals and organisations with your institution as well as using disclosure reports comprising payments to healthcare professionals and organisations published on their individual websites)?
19. The EFPIA code of practice covers the category of payments related to research and development. Are these payments disclosed by your institution on a named basis or on drug company websites, in line with the EFPIA code of practice?

If payments made in 2019 calendar were reported year please could you answer the following questions regarding payments from <u>all industries</u> whose payments are managed by your institution?

20. How many companies reported payments in 2019?
21. What was the overall number of healthcare professionals receiving payments in 2019?
22. (If relevant) What was the overall number of healthcare organisations receiving payments in 2019?
23. (If relevant) What was the overall number of patient organisations receiving payments in 2019?
24. What was the overall value of payments made to healthcare professionals in 2019?
25. (If relevant) What was the overall value of payments made to healthcare organisations in 2019?
26. (If relevant) What was the overall value of payments made to patient organisations in 2019?

Please could you answer the following questions regarding only payments made by <u>pharmaceutical companies only</u> in the 2019 calendar year?

27. How many pharmaceutical companies reported payments in 2019?
28. Is the number of companies which did not disclose any payments made in 2019 known? If so, please could you state this number?
29. What was the overall number of healthcare professionals receiving payments from pharmaceutical companies in 2019?
30. (If relevant) What was the overall number of healthcare organisations receiving payments receiving payments from pharmaceutical companies in 2019?
31. (If relevant) What was the overall number of patient organisations receiving payments receiving payments from pharmaceutical companies in 2019?
32. What was the overall share of healthcare professionals consenting to their payments being disclosed in 2019?
33. (If relevant) What was the overall share of healthcare organisations consenting to their payments being disclosed in 2019?
34. (If relevant) What was the overall share of patient organisations consenting to their payments being disclosed in 2019?
35. What was the overall value of all payments made to healthcare professionals by pharmaceutical companies in 2019?
36. (If relevant) What was the overall value of payments made to healthcare organisations by pharmaceutical companies in 2019?
37. (If relevant) What was the overall value of payments made to patient organisations by pharmaceutical companies in 2019?

38. Please could you provide the value of each category of payments received by healthcare professionals from pharmaceutical companies in 2019?
39. (If relevant) Please could you provide overall the value of each category of payments received by healthcare organisations from pharmaceutical companies in 2019?
40. (If relevant) Please could you provide overall the value of each category of payments received by patient organisations from pharmaceutical companies in 2019?

Finally, I would be grateful if you could answer a few general questions regarding the direction nature of payments disclosed.

41. The obligatory disclosure of payments by a public body, such as your institution, seem be at odds the approach taken by many other European countries, which support a self-regulatory system managed by the pharmaceutical industry and allowing payment recipients not to have their payments disclosed based on the General Data Protection Regulation (GDPR). How does the system for payment disclosure managed by your institution addresses potential concerns regarding data privacy of payment recipients?
42. What are the advantages of the mandatory disclosure of payments, overseen by your institution, over a self-regulatory payment system based on the EFPIA Code of practice, existing in many other European countries?
43. What is the source of funding of the disclosure system managed by your institution (e.g. general taxation, health insurance, company fees)?
44. What is the yearly cost of maintaining the disclosure system managed by your institution?
45. How there been any examples of healthcare professionals not complying with the requirements of the disclosure system managed by your institution? If so, how were they addressed?
46. (if relevant) How there been any examples of healthcare organisations not complying with the requirements of the disclosure system managed by your institution? If so, how were they addressed?
47. (if relevant) How there been any examples of patient organisations not complying with the requirements of the disclosure system managed by your institution? If so, how were they addressed?
48. How there been any examples of pharmaceutical companies not complying with the requirements of the disclosure system managed by your institution? If so, how were they addressed?
49. Does your institution monitor who uses the disclosed payment data? If so, would you be able to say who the key types of users are?
50. What does your institution do to encourage the use of disclosed data (e.g. public campaigns)?
