# Supplementary material for: Tip of the Iceberg? Country- and Company-Level Analysis of Drug Company Payments for Research and Development in Europe
Source: Int J Health Policy Manag. 2022 Mar 15;11(12):2842–59. doi: 10.34172/ijhpm.2022.6575 (PMC10105170; doi:10.34172/ijhpm.2022.6575)
Supplement: Supplementary file 2 — Background Data.81,93-95,126-134,136,187-190 [file ijhpm-11-2842-s002.pdf]

Article title: Tip of the Iceberg? Country- and Company-Level Analysis of Drug Company Payments for Research and Development in Europe

Journal name: International Journal of Health Policy and Management (IJHPM)

Authors' information: Piotr Ozieranski[1]*, Luc Martinon[2], Pierre-Alain Jachiet[2], Shai Mulinari[3]

[1]Department of Social and Policy Sciences, University of Bath, Bath, UK.

[2]Euros for Docs, Paris, France.

[3]Department of Sociology, Lund University, Lund, Sweden.

(*Corresponding authors: p.ozieranski@bath.ac.uk)

Supplementary file 2. Background Data

Table S1: Distribution of drug company payments in European countries pursuing self-regulation of payment disclosure

	**Total[2]**	**Joint working[3]**		**Donations and grants**		**Fees for services & consultancy**		**Contributions to costs of events**		**R&D**	
**Country[1]**	**€m[4]**	**€m[4]**	**%**	**€m[4]**	**%**	**€m[4]**	**%**	**€m[4]**	**%**	**€m[4]**	**%**
UK	619	5	1%	50	8%	73	12%	55	9%	435	70%
GERMANY	629									404	64%
SPAIN	601			34	6%	84	14%	224	37%	259	43%
BELGIUM[5]	239			22	9%	21	9%	43	18%	154	64%
THE NETHERLANDS[5, 6]	199			N/A	N/A	N/A	N/A	N/A	N/A	135	68%
POLAND	164			9	6%	17	10%	33	20%	106	64%
SWEDEN	90			1	1%	10	11%	5	5%	73	82%
SWITZERLAND	167									62	37%
AUSTRIA	134			6	5%	16	12%	53	39%	59	44%
FINLAND	40									25	63%
IRELAND	35			3	9%	2	6%	9	26%	21	59%
ROMANIA[7,8]	48									20	42%
NORWAY	19									13	68%
CZECH REPUBLIC											70%
**Total**	**2,985**									**1,766**	

Notes.

[1] – Data sources:

- Austria,[127] Belgium,[189] Germany,[128] Poland,[129] Spain,[130] the Netherlands,[131] and Switzerland[132] – publicly available national pharmaceutical industry group press releases.
- Czech Republic – the EFPIA 2019 Europe-wide report.[82]
- Finland,[133] Norway,[134] and Sweden[135] – email communication with the national pharmaceutical industry trade groups.
- Ireland – the EFPIA 2019 Europe-wide report[82] and email communication with the national pharmaceutical industry trade group.[190]
- Romania – a national pharmaceutical industry trade group report,[94] individual drug company websites signposted in the report, and data obtained from the public database of industry payments.[96]
- UK – a trade group press release[191] as well as calculations using data obtained from Disclosure UK, a publicly available database of pharmaceutical industry payments.[95]
- If more than one data source was available data provided directly by pharmaceutical industry trade groups took precedence over national-level reports, which, in turn, took precedence over the EFPIA Europe-wide report.

[2] – Given the rounding the totals do not always match the sum of payments related to different payment categories.

[3] – Joint working is a unique payment category introduced only by the Association of the British Pharmaceutical Industry. It covers projects involving funders and recipients pulling together resources to deliver shared projects.[192]

[4] – All payment values in non-euro currencies were converted to euros based on the average yearly exchanged rates published by the European Central Bank.[137]

[5] – All statistics for Belgium and the Netherlands cover both the pharmaceutical and medical devices industries. Statistics for other countries cover the pharmaceutical industry only.

[6] – Non-R&D payments reported in the Netherlands do not follow the EFPIA Code, but R&D payments are reported consistently with the EFPIA Code, including their non-disclosure on a named basis. This means R&D payments reported in the Netherlands can be added to R&D payments reported in the other countries reported in the table. In addition, the sum of non-R&D payments reported in the Netherlands was obtained by adding payments made either to healthcare professionals or organisations. Therefore, the sum of payments reported in the Netherlands can be compared with the other country sums from the table.

[7] – The total value of industry payments in Romania in 2019 includes €28m of non-R&D payments (calculated based on data reported in a publicly-run disclosure platform) and €20m of R&D payments

(calculated using a pharmaceutical industry trade group report and data from individual drug company websites – see footnote 1).

[8] – The value of R&D payments in Romania was calculated using a report published on the website of the Romanian pharmaceutical industry trade group[94] and individual drug company websites mentioned in the report.

Table S2. Top 20 drug company funders in Belgium, Ireland and the UK based on the value of R&D payments (2017-19)

	BELGIUM					IRELAND					UK				
	**Company name**	**R&D payments – value (€m)**	**At least once in global top 50**	**Global sales – value (€m)**	**Global sales – rank**	**Company name**	**R&D payments – value (€m)**	**At least once in global top 50**	**Global sales – value (€m)**	**Global sales – rank**	**Company name**	**R&D payments – value (€m)**	**At least once in global top 50**	**Global sales – value (€m)**	**Global sales – rank**
1	Roche	55	Yes	117,882	1	Allergan	16	Yes	39,219	17	Astrazeneca	167	Yes	55801	12
2	Msd	43	Yes	99,576	4	Abbvie	4	Yes	80,690	7	Allergan	102	Yes	39219	17
3	Janssen	36	Yes	99,220	5	Janssen	4	Yes	99,220	5	Bristol-Myers-Squibb	93	Yes	71725	9
4	Astrazeneca	28	Yes	55,801	12	Msd	4	Yes	99,576	4	Roche	76	Yes	117882	1
5	Novartis	24	Yes	115,170	3	Bristol-Myers-Squibb	3	Yes	71,725	9	Novartis	73	Yes	115170	3
6	Celgene	19	Yes	24,383	24	Pfizer	3	Yes	117,627	2	Bayer	71	Yes	47632	15
7	Pfizer	18	Yes	117,627	2	Servier	2	Yes	12,543	30	Msd	70	Yes	99576	4
8	Bristol-Myers-Squibb	17	Yes	71,725	9	Bayer	2	Yes	47,632	15	Janssen	69	Yes	99220	5
9	Boehringer-Ingelheim	12	Yes	39,187	18	Novartis	2	Yes	115,170	3	Pfizer	64	Yes	117627	2
10	Bayer	12	Yes	47,632	15	Roche	2	Yes	117,882	1	Glaxosmithkline	63	Yes	79356	8

11	Servier	10	Yes	12,543	30	Gilead	2	Yes	60,525	10	Celgene	45	Yes	24383	24
12	Abbvie	10	Yes	80,690	7	Shire	1	Yes	25,524	23	Boehringer-Ingelheim	32	Yes	39187	18
13	Lilly	9	Yes	50,978	14	Astrazeneca	1	Yes	55,801	12	Ucb	27	Yes	13023	29
14	Medtronic	8	No	0	-	Amgen	1	Yes	58,268	11	Biogen	26	Yes	28580	22
15	Glaxosmithkline	8	Yes	79,356	8	Glaxosmithkline	1	Yes	79,356	8	Servier	25	Yes	12543	30
16	Regeneron-Ireland-Unlimited-Company	7	Yes	11,097	35	Celgene	1	Yes	24,383	24	Takeda	25	Yes	52943	13
17	Amgen	6	Yes	58,268	11	Novo-Nordisk	1	Yes	46,423	16	Novo-Nordisk	24	Yes	46423	16
18	Gilead	5	Yes	60,525	10	Biogen	0	Yes	28,580	22	Sanofi	20	Yes	91197	6
19	Sanofi	4	Yes	91,197	6	Ucb	0	Yes	13,023	29	Amgen	20	Yes	58268	11
20	Amplitude	3	No	0	-	Boehringer-Ingelheim	0	Yes	39,187	18	Abbvie	19	Yes	80690	7

Table S3: Top 20 drug company funders in Belgium, Ireland and the UK based on the value of all payments (2017-19)

	BELGIUM					IRELAND					UK				
	**Company name**	**R&D payments – value (€m)**	**At least once in global top 50**	**Global sales – value (€m)**	**Global sales – rank**	**Company name**	**R&D payments – value (€m)**	**At least once in global top 50**	**Global sales – value (€m)**	**Global sales – rank**	**Company name**	**R&D payments – value (€m)**	**At least once in global top 50**	**Global sales – value (€m)**	**Global sales – rank**
1	Roche	69	Yes	117,882	1	Allergan	16	Yes	39,219	17	Astrazeneca	183	Yes	55,801	12
2	Msd	56	Yes	99,576	4	Abbvie	9	Yes	80,690	7	Allergan	107	Yes	39,219	17
3	Janssen	52	Yes	99,220	5	Pfizer	7	Yes	117,627	2	Glaxosmithkline	106	Yes	79,356	8
4	Astrazeneca	34	Yes	55,801	12	Janssen	7	Yes	99,220	5	Novartis	103	Yes	115,170	3
5	Novartis	32	Yes	115,170	3	Novartis	6	Yes	115,170	3	Bristol-Myers-Squibb	102	Yes	71,725	9
6	Pfizer	31	Yes	117,627	2	Msd	6	Yes	99,576	4	Pfizer	101	Yes	117,627	2
7	Celgene	22	Yes	24,383	24	Bristol-Myers-Squibb	5	Yes	71,725	9	Bayer	100	Yes	47,632	15
8	Bayer	20	Yes	47,632	15	Bayer	4	Yes	47,632	15	Roche	91	Yes	117,882	1
9	Bristol-Myers-Squibb	20	Yes	71,725	9	Roche	4	Yes	117,882	1	Janssen	87	Yes	99,220	5
10	Boehringer-Ingelheim	19	Yes	39,187	18	Servier	3	Yes	12,543	30	Msd	84	Yes	99,576	4

11	Medtronic	18	No	-	-	Gilead	3	Yes	60,525	10	Celgene	50	Yes	24,383	24
12	Abbvie	16	Yes	80,690	7	A-Menarini	2	Yes	8,421	41	Novo-Nordisk	47	Yes	46,423	16
13	Glaxosmithkline	15	Yes	79,356	8	Sanofi	2	Yes	91,197	6	Boehringer-Ingelheim	40	Yes	39,187	18
14	Lilly	13	Yes	50,978	14	Astrazeneca	2	Yes	55,801	12	Sanofi	37	Yes	91,197	6
15	Amgen	13	Yes	58,268	11	Amgen	2	Yes	58,268	11	Biogen	36	Yes	28,580	22
16	Gilead	12	Yes	60,525	10	Novo-Nordisk	2	Yes	46,423	16	Takeda	34	Yes	52,943	13
17	Servier	12	Yes	12,543	30	Glaxosmithkline	2	Yes	79,356	8	Abbvie	34	Yes	80,690	7
18	Sanofi	9	Yes	91,197	6	Shire	2	Yes	25,524	23	Ucb	33	Yes	13,023	29
19	St.-Jude-Medical-Belgium	9	No	-	-	Ucb	1	Yes	13,023	29	Lilly	32	Yes	50,978	14
20	Merck	8	Yes	18,758	27	Astellas	1	Yes	29,236	21	Gilead	29	Yes	60,525	10

Table S4: Top 20 drug company funders in Belgium, Ireland and the UK based on R&D payments as a share of all payments (2017-19)

	**BELGIUM**					**IRELAND**					**UK**				
	**Company name**	**Share of R&D payments**	**At least once in global top 50**	**Global sales – value (€m)**	**Global sales – rank**	**Company name**	**Share of R&D payments**	**At least once in global top 50**	**Global sales – value (€m)**	**Global sales – rank**	**Company name**	**Share of R&D payments**	**At least once in global top 50**	**Global sales – value (€m)**	**Global sales – rank**
1	Thrombogenics	0.98	No	-	-	Allergan	1.00	Yes	39,219	17	Clinuvel	1.00	No	-	-
2	Regeneron-Ireland-Unlimited-Company	0.98	Yes	11,097	35	Servier	0.76	Yes	12,543	30	Biotest	0.98	No	-	-
3	Sarepta	0.97	No	-	-	Swedish-Orphan-Biovitrum	0.73	No	-	-	Bluebird	0.97	No	-	-
4	Amplitude	0.94	No	-	-	Gilead	0.70	Yes	60,525	10	Allergan	0.95	Yes	39,219	17
5	Hra	0.92	No	-	-	Celgene	0.69	Yes	24,383	24	Otsuka	0.94	Yes	15,571	28
6	Jazz	0.88	No	-	-	Shire	0.68	Yes	25,524	23	Diurnal	0.94	No	-	-
7	Zoetis-Belgium	0.87	No	-	-	Bristol-Myers-Squibb	0.66	Yes	71,725	9	Astrazeneca	0.91	Yes	55,801	12
8	Celgene	0.85	Yes	24,383	24	Msd	0.64	Yes	99,576	4	Bristol-Myers-Squibb	0.91	Yes	71,725	9
9	Cochlear-Benelux	0.84	No	-	-	Janssen	0.63	Yes	99,220	5	Shionogi	0.91	No	-	-

10	Bellco	0.84	No	-	-	Boehringer-Ingelheim	0.51	Yes	39,187	18	Celgene	0.91	Yes	24,383	24
11	Astrazeneca	0.83	Yes	55,801	12	Bayer	0.47	Yes	47,632	15	Eisai	0.89	Yes	11,909	31
12	Nevro-Medical	0.82	No	-	-	Abbvie	0.47	Yes	80,690	7	Servier	0.88	Yes	12,543	30
13	Bristol-Myers-Squibb	0.82	Yes	71,725	9	Biogen	0.45	Yes	28,580	22	Biomarin	0.88	No	-	-
14	Otsuka	0.82	Yes	15,571	28	Glaxosmithkline	0.44	Yes	79,356	8	Gw-Pharmaceuticals	0.87	No	-	-
15	Servier	0.80	Yes	12,543	30	Roche	0.43	Yes	117,882	1	Indivior	0.86	No	-	-
16	Roche	0.80	Yes	117,882	1	Amgen	0.42	Yes	58,268	11	Tesaro	0.86	No	-	-
17	Duo---Med	0.79	No	-	-	Astrazeneca	0.41	Yes	55,801	12	Msd	0.83	Yes	99,576	4
18	Msd	0.76	Yes	99,576	4	Pfizer	0.41	Yes	117,627	2	Clovis-Oncology	0.83	No	-	-
19	Novartis	0.73	Yes	115,170	3	Almirall	0.38	No	-	-	Roche	0.83	Yes	117,882	1
20	Volcano-Europe	0.70	No	-	-	Novo-Nordisk	0.34	Yes	46,423	16	Mitsubishi	0.81	Yes	7,327	43

Table S5: Shared companies in the top 20 drug company funders in Belgium, Ireland and the UK based (2017-19)

	**BELGIUM – IRELAND**	**BELGIUM – UK**	**IRELAND – UK**
R&D payments	0.75	0.75	0.90
All payments	0.70	0.75	0.75
Share of R&D payments	0.30	0.35	0.35
